# Pre-Disease and Pre-Surgery BMI, Weight Loss and Sarcopenia Impact Survival of Resected Lung Cancer Independently of Tumor Stage

**DOI:** 10.3390/cancers12020266

**Published:** 2020-01-22

**Authors:** Philippe Icard, Olivier Schussler, Mauro Loi, Antonio Bobbio, Audrey Mansuet Lupo, Marie Wislez, Antonio Iannelli, Ludovic Fournel, Diane Damotte, Marco Alifano

**Affiliations:** 1 Thoracic Surgery Department, Paris Center University Hospitals, AP-HP, 75014 Paris, France; phlippe.icard@aphp.fr (P.I.); olivier.schussler@aphp.fr (O.S.); antonio.bobbio@aphp.fr (A.B.); ludovic.fournel@aphp.fr (L.F.); 2 Medical School, Inserm UMR 1199, Biology and Innovative Therapies of Locally Advanced Cancers University Caen Normandie, 14032 Caen, France; 3 Department of Radiation Oncology, Humanitas Hospital, Milan 20089, Italy; mauro.loi@humanitas.it; 4 Pathology Department, Paris Center University Hospitals, AP-HP, 75014 Paris, France; audrey.lupo@aphp.fr (A.M.L.); diane.damotte@aphp.fr (D.D.); 5 Inserm U1138, Integrative Cancer Immunology, University Paris Descartes, 75006 Paris, France; marie.wislez@aphp.fr; 6 Thoracic Oncology Unit, Paris Center University Hospitals, AP-HP, 75014 Paris, France; 7 Department of Digestive Surgery and Liver Transplantation, Nice University Hospital, 06000 Nice, France; antonio.iannelli@unice.fr; 8 Department of nutrition, University of Nice Côte d’Azur, 06000 Nice, France; 9 Inserm UMR-S 1124, Cellular Homeostasis and Cancer, Paris-Descartes University, 75006 Paris, France

**Keywords:** lung cancer, surgery, outcome, body mass index, weight loss, sarcopenia, morphomics

## Abstract

Lower pre-surgery Body Mass Index (BMI) and low muscle mass impact negatively long-term survival of non-small cell lung cancer (NSCLC). We investigated their influence on survival after major lung resection for NSCLC. Methods: A retrospective analysis of a prospectively collected database was made on 304 consecutive patients. Results: Underweight, normal, overweight and obese patients represented 7.6%, 51.6%, 28.6%, and 12.6% of the pre-disease population. Weight loss and gain were recorded in 44.4% and 5% of patients, respectively. Low muscle mass was more frequently associated with BMI < 25 kg/m^2^ (*p* < 0.000001). Overall survival was positively affected by pre-disease (*p* = 0.036) and pre-surgery (*p* = 0.017) BMI > 25 kg/m^2^, and, even more, in case of BMI > 25 kg/m^2^ and increasing weight (*p* = 0.012). Long-term outcome was negatively influenced by low muscle mass (*p* = 0.042) and weight loss (*p* = 0.0052) as well as age (*p* = 0.017), ASA categories (*p* = 0.025), extent of resection (*p* = 0.0001), pleural invasion (*p* = 0.0012) and higher pathologic stage (*p* < 0.0001). Three stepwise multivariable models confirmed the independent favorable prognostic value of higher pre-disease (RR 0.66[0.49–0.89], *p* = 0.006) and pre-surgery BMI (RR 0.72[0.54–0.98], *p* = 0.034), and the absence of low muscle mass (RR 0.56[0.37–0.87], *p* = 0.0091). Conclusions: Body reserves assessed by simple clinical markers impact survival of surgically treated NSCLC. Strategies improving body fat and muscular mass before surgery should be considered.

## 1. Introduction

In contrast to the widely accepted idea that higher Body Mass Index (BMI) decreases the survival of many malignant tumors (such as colonic, pancreatic and post-menopausal breast cancers) and increases their incidence [1], obesity (defined as BMI >30 kg/m^2^) would be protective for survival and/or occurrence of few malignancies, including large B-cell lymphomas, renal, and non-small cell lung cancer (NSCLC) [2]. This “obesity paradox” has been suggested by epidemiological studies [3] and recent meta-analysis dealing particularly with NSCLC [4,5]. However, these studies were unable to determine if these differences in survival are really due to BMI or to possibly related confounding factors, such as the extent of disease which was most often not recorded. However, nutritional status assessed by measurement of plasmatic markers has been recognized in recent years as an important determinant of survival in operated NSCLC [6]. Moreover, sarcopenia [7] and lower BMI [8,9] have been recognized to increase post-operative morbidity and/or mortality. A recent study by our group showed that pre-surgery BMI > 25 kg/m^2^ impacts favorably long-term survival of patients who had undergone pneumonectomy for NSCLC, independently of stage [10]. This favorable influence of higher BMI is confirmed in obese patients who had undergone lobectomy for cancer [11]. Considering that weight loss (WL) significantly worsens survival of advanced NSCLC [12], the negative prognostic impact of lower BMI [10] could simply reflect the consequence of WL. Thus, for better understanding the impact of BMI on outcome, it should be useful to study, together with pre-surgery BMI, pre-disease BMI, in the idea that baseline reserves could by themselves impact survival. Indeed, BMI and sarcopenia can be considered as good indicators of fat storage and muscular loss, respectively [13]. Sarcopenia is associated with weakness, frailty, aging, various chronic diseases such as Chronic Obstructive Pulmonary Disease (COPD) and advanced cancers [14,15,16,17]. It is also predictive of reduced long-term outcome after pneumonectomy [10]. According to updated definition, sarcopenia is now defined as a muscle disease that can be confirmed through quantification of muscle mass and whose severity correlates with loss of strength; low muscle mass has also been proposed as part of the definition of malnutrition [17]. However, sarcopenia is matter of debate about definition, calculation and cut-off points, and above all, methods of measurements. Indeed, current imaging methods (CT scan and magnetic resonance imaging, MRI) have been validated by few studies demonstrating correlation between cross regional muscle mass measurement with whole-body muscle mass calculation, as recently reviewed [18,19]. Thus, some authors [2,19] have pointed out the need to compare imaging methods with the classical clinical assessment of sarcopenia derived from the calculation of height indexed total muscular mass (iTMM) by Heymsfield’ formulas [20]. This anthropometric method has been long considered robust as validated by studies on cadaver or on bioelectrical impedance analysis (BIA), dual-energy X-ray absorptiometry (DEXA), CT and MRI determinations [20,21,22,23,24]. Very recently, Park et al. also outlined the great heterogeneity and limited sample sizes of available imaging studies on sarcopenia, the lack of proper control of confounders with most often no references to WL, stage of disease and therapy [2]. Thus, the objective of our study was to assess, in 304 consecutive patients who underwent resection of NSCLC: (1) the relationships between clinically recorded morphometric parameters (pre-disease and pre-surgery BMI, WL and iTTM) and main usual clinical and pathological parameters; (2) their respective impact on long-term survival.

## 2. Results

### 2.1. Clinical and Morphometric Features

Three-hundred and four patients underwent surgery for NSCLC in the study period. Their demographic, clinical, and pathologic parameters are detailed in Table 1. As usually, patients were more frequently men, with current or past history of tobacco smoking and chronic bronchitis, and who underwent lobectomy for stage I–II disease.

Median pre-surgery BMI was 24.3 kg/m^2^ (21.4–26.8], with underweight, normal-weight, overweight and obese patients representing 7.4% (*n* = 23), 50.3% (*n* = 157), 27.9% (*n* = 87), and 11.8% (*n* = 37) respectively.

Median pre-disease BMI was 24.9 kg/m^2^ (22.5–27.3). Underweight, normal-weight, overweight and obese patients (on the basis of pre-disease self-reported weight) represented 3.8% (*n* = 12), 46.1% (*n* = 144), 34.6% (*n* = 108), and 12.8% (*n* = 40), respectively.

Median percent weight change was 0 (0–6.75). In total, 153 patients (50.3%) had stable body weight in the 6 months prior to surgery; 135 (44.4%) patients reported WL and 16 (5.2%) weight increase (WI) (Table 1). Of note, WI was observed mainly (10/16) in obese patients. WL < 5%, between 6%–10%, and >10% of pre disease body weight was recorded in 34.8% (47/135), 36.3% (49/135), and 28.8% (39/135) of patients, respectively.

The correlations between morphometry parameters and clinical and pathological factors are shown in Table 2. BMI > 25 Kg/m^2^ was more frequently associated with male sex, advanced age and lower disease stage.

Mean iTMM (available for 147 patients) was 7.3 + 1.7 kg/m^2^; 6.5 kg/m^2^ represented the 33rd percentile and was set as the cut-off to indicate low muscle mass and used for subsequent analyses. According to weight distribution, low muscle mass was recorded as follows: 66% in underweight, 39% in normal weight, 21% in overweight and 9.5% in obesity (*p* = 0.0021).

iTMM was correlated with pre-surgery BMI (*r* = 0.537, *p* < 0.0000001). Median pre-surgery BMI was 21.4 kg/m^2^ (19.0–24.3) and 24.9 kg/m^2^ (22.4–29.6) in the presence of low and normal muscle mass, respectively (*p* < 0.0000001).

iTMM was inversely correlated with weight loss (*r* = −0.247, *p* = 0.0028). Median weight loss was 3.0 kg (0–10) and 0 kg (0–4.5) in presence of low and normal muscle mass, respectively (*p* = 0.034).

iTMM was also directly correlated with pre-disease BMI (*r* = 0.512, *p* < 0.0000001). Median pre disease BMI were 22 kg/m^2^ (20.3–24.5) and 26 kg/m^2^ (23.5–29) in presence of low and normal muscle mass respectively (*p* < 0.0000001).

### 2.2. Long-Term Outcome

The median survival of the whole population was 52 months. Three, 5 and 7-year survival rates were 57.9%, 46.5%, and 38.4%, respectively. Five-year survival after lobectomy and pneumonectomy was 52.9% (46.4–59.2) and 28.2% (19.0–39.5), respectively.

Table 3 shows the results of univariable survival analysis demonstrating the significant impact of following usual clinical and pathological parameters: age (*p* = 0.017), ASA (American Society of Anesthesiologists classification of physical status) categories (*p* = 0.025), extent of resection (*p* = 0.0001), pleural invasion (*p* = 0.0012) and pathologic stage (*p* < 0.0001). As expected, low muscle mass also negatively impacted the 5-year survival rates (*p* = 0.042). Five-year survival rates were 48.9% and 56.6% in the presence of low and normal muscle mass, respectively; differences even more pronounced at 7 years, with figures of 31.6 and 50.1%, respectively (Table 4).

**Figure 1:**
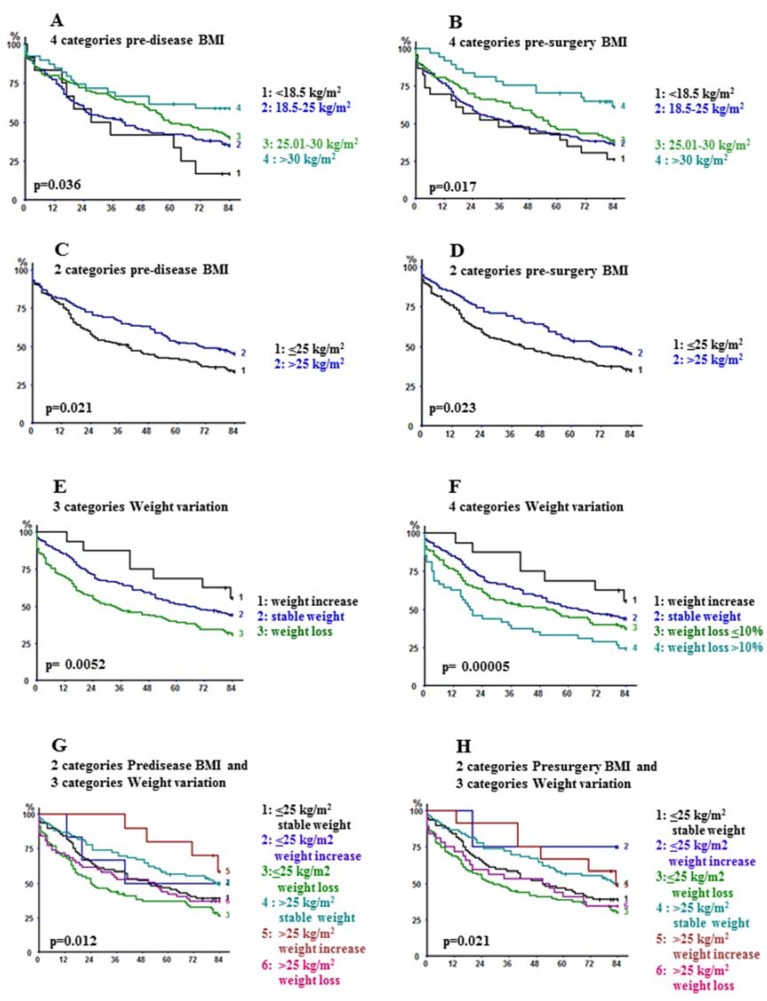
Kaplan–Meier overall survival analyses and log-rank comparisons with respect to: (**A**) pre-disease BMI (four categories: underweight, normal weight, overweight, obesity); (**B**) pre-surgery BMI (four categories); (**C**) pre-disease BMI (two categories: underweight-normal weight versus overweight-obesity); (**D**) pre-surgery BMI (two categories); (**E**) weight variation (three categories: stable weight, weight loss, weight increase); (**F**) weight variation (4 categories: stable weight, weight increase, weight loss <10%, weight loss >10%); (**G**) two categories pre-disease BMI and three categories weight variation (six groups), (**H**) two categories pre-surgery BMI and three categories weight variation (six groups).

Pre-disease (*p* = 0.036) and pre-surgery BMI (*p* = 0.017) predicted long-term outcome (Table 4; Figure 1A,B). Because of slight differences in survival between underweight and normal-weight individuals with respect to both pre-disease and pre-surgery BMI, we pooled underweight and normal-weight on one side and overweight and obesity on the other (Figure 1C, D). Survivals were significantly different (*p* = 0.023), with 5-year figures of 42.2% and 53.3% in patients with pre-surgery BMI < 25 kg/m^2^ and >25 kg/m^2^ respectively (Table 4). Similarly, with respect to pre-disease BMI, these figures were 42% and 52.4% in patients with BMI < 25 kg/m^2^ and >25 kg/m^2^ (log-rank *p* = 0.021), respectively. Among patients with BMI > 25, those with obesity (BMI > 30) had significantly better outcome than those with overweight (BMI between 25 and 30) both evaluated on pre-disease (*p* = 0.049) or pre-surgery (*p* = 0.017) weight.

Univariable analysis also showed the favorable impact of WI, as compared to stable weight and WL (*p* = 0.0052) (Figure 1E), particularly when it exceeded 10% (*p* = 0.00005) (Figure 1F).

Further survival analyses were performed with respect to weight change (stable, increase, loss) in patients with pre-disease BMI > 25 kg/m^2^ on the one side and <25 kg/m^2^ on the other one (six groups, *p* = 0.012) (Figure 1G). Globally, four distinct subsets of patients could be identified according to increasing worsening of outcome: (1) those with BMI > 25 kg/m^2^ and WI; (2) those with BMI > 25 kg/m^2^ and stable weight or those with BMI < 25 kg/m^2^ and WI; (3) those with BMI < 25 kg/m^2^ and stable weight or with BMI >25 kg/m^2^ and WL; and (4) those with BMI < 25 kg/m^2^ and WL. Similar analyses performed on the basis of pre-surgery BMI demonstrated super-posable results (*p* = 0.021) (Figure 1H).

### 2.3. Multivariable Analysis

Because of the significant interactions between covariates, we built three stepwise multivariable models (Table 5), including, respectively, pre-disease BMI, pre-surgery BMI and iTMM. All these models included the clinical and pathologic parameters identified at univariable analysis as associated with survival (age, ASA class, extent of resection, stage of disease) together with the respective factor related to morphomics. All three models confirmed, together with stage of disease and ASA class, the independent favorable prognostic value of higher pre-disease (RR 0.66 (0.49–0.89), *p* = 0.006), higher pre-surgery BMI (RR 0.72 (0.54–0.98), *p* = 0.034) and of the absence of low muscle mass (RR 0.56 (0.37–0.87), *p* = 0.0091).

## 3. Discussion

Our study collected clinical, morphomic and pathological data of a large series of 304 consecutive patients undergoing major resection for NSCLC. Because our study was a retrospective analysis of a prospectively collected database, few data were missing. Its main limitation relies in the retrospective collection of long-term outcome parameters, preventing calculation of disease-specific survival, and allowing only calculation of overall survival. It should be pointed out that pre-surgery BMIs were determined by body weight measurements routinely performed at our institution as opposed to BMIs extrapolated from patient histories; anyway, it is our feeling that this difference in data collection may significantly affect the results. Furthermore, adjuvant treatments were not standardized. However, these two parameters very likely did not significantly affect the results.

The median pre-disease BMI recorded in current series (24.9 kg/m^2^) was identical to the median BMI of French general population. Our percent of underweight (<10%), normal weight (50%), and overweight-obese patients (around 40%) were also almost identical to those recorded in a Canadian report [25], as well as in a large French cohort of 6595 patients diagnosed with NSCLC in several non-academic centers [26].

### 3.1. The Obesity Paradox Is a Reality in Resectable NSCLC

Our study shows that BMI impacts survival independently of TNM: patients with pre-surgery BMI <25 kg/m^2^ or >25 kg/m^2^ had a significant difference in survival, reaching around a 10% difference in 5-year figures (42.2% vs. 53.3%). It is noteworthy that the “obesity paradox” of NSCLC may apply also to lung cancer risk occurrence [27], possibly due to the lower concentration of polycyclic aromatic hydrocarbons in lung cells due to carcinogen molecules retention in fat tissue [28].

### 3.2. Lower BMI, Weight Loss and Low Muscle Mass Negatively Impact the Long-Term Outcome

Weight loss is notably related to morbidity/mortality in hospitalized patients, especially if surgically treated [9,16,29]. It is also a predictive factor of shorter survival in patients with advanced inoperable lung cancers [12,26]. Schematically, almost half of our patients (44.4%) showed WL. As we have shown, WL impacts the long-term survival of all patients, independently of TNM, and particularly of those with a lower BMI [24]. Patients with WL > 10% have a significantly lower survival than those with less pronounced WL [29]. Our results confirm few recent studies reporting the negative impact of low muscle mass on survival of operated lung cancers [10,30]. Low muscle mass was mostly observed in underweight and normal weight patients (66% and 39% respectively), less frequently in overweight (21%), and rarely in obese patients (9.5%). This scarcity of sarcopenic obesity (SO) in stage I–III patients is in agreement with the report of Prado et al. [31] which recorded 8% (20/250) of SO in a subpopulation of non-metastatic cancer patients, much lower than figures (around 25%) usually recorded in advanced and metastatic disease, where low muscle mass is included in a process of cachexia announcing poor survival [15,16]. It is noteworthy that muscle loss occurring in follow-up negatively impacts survival of early-stage resected NSCLC in an independent manner [32].

### 3.3. The Difficulties of Standard Assessment of Low Muscle Mass

Our cut-off of 6.5 kg/m^2^ defining low muscle mass as a loss of height-normalized total muscular mass below the 33rd percentile was in agreement with the current literature [14,17]. However, muscle mass is often extrapolated from cross-sectional skeletal muscle area (SMA) determined by CT scan at the 12th thoracic or L3 vertebra by using a single slice. This measurement expressed as cm^2^ is frequently adjusted to square height and results expressed in skeletal muscle index (SMI, cm^2^/m^2^) [33]. Thus, it is difficult to compare series where low muscle mass is calculated by using different parameters and/or cut-offs including total psoas area or SMA or SMI, with considerable variation in the modalities of imaging assessment [34]. Furthermore, the comparison of dual-energy X-ray absorptiometry (DEXA measurements) with bioimpedance analysis (BIA) and/or with CT/MRI, relying on different physical parameters, make interpretation and correction of discordances quite impossible [35]. Several authors [2,13,16,19,34] have recently outlined the need to reconsider standard clinical assessment of low muscle mass: it is urgent to develop consensus criteria, and in particular, to establish the accuracy of CT-based evaluation [2,19,34]. There is also an urgent need to integrate functional measurements (strength or performance) of low muscle mass to morphologic ones [13,17].

### 3.4. Understanding Why Pre-Operative Nutritional Status Impacts on Survival

As we have seen, high and/or stable pre-disease BMI positively influences survival of NSCLC, in contrast to low pre-disease BMI, WL and low muscle mass. The explanation could rely on both a special metabolism of lung tumors and/or of the host, as well as on stimulation of immune defense. In that view, the lack of body reserves occurring in cachexia promotes a catabolic state favoring cancer development [36]. Furthermore, it is known that malnutrition is a major cause of immunodeficiency promoting infection and cancer development. Thus, it is tempting to conclude that high fat may promote a good state of immune defense against NSCLC, delaying cancer cells development. We previously reported in resected NSCLC that tumor infiltrating immune cells, in particular cytotoxic CD8+ lymphocytes, was directly correlated with good nutritional status and was finally associated with better survival [6]. We can hypothesize that the function of these cytotoxic CD8+ lymphocytes is promoted by overweight/obesity in NSCLC, in contrast to many others cancers where obesity is associated with exhaustion of CD8+ lymphocytes [37]. Further studies will be necessary in NSCLC to study if obesity impacts expression of PD-1, exhaustion of CD8+ T lymphocytes, or absence of CD39 in these cells indicating bystander lymphocytes not stimulated by neoantigens [38].

From a metabolic point of view, lack of body reserve may promote a catabolic state of the host, resulting in the enhancement of liver gluconeogenesis providing glucose for tumor consumption. Gluconeogenesis is sustained by glycerol derived from lipolysis, a process sparing and delaying the consumption of amino-acids derived from proteolysis [39]. Thus, the consumption of fat storages sparing proteins would attenuate the loss of skeletal muscle. However, low-fat reserves force cancer cells to consume more and more amino-acids in a vicious cycle promoting finally low muscle mass, WL and altered immune response. This perverted metabolism of the host would produce glucose for proliferation of cancer cells, especially relying on aerobic glycolysis (i.e., the Warburg effect), associated with poor differentiation and survival [40,41,42]. The Warburg effect alters immune response, in particular by various actions of lactate secreted by cancer cells [43], and because of the preferential glucose uptake by cancer cells preventing activation of cytotoxic cells [44]. Understanding cancer cells metabolism in relation with microenvironment contexture, nutrients intake, mitochondrial functioning, cell differentiation, organ distribution of metastases, inflammation and metabolic messengers, is a new fascinating area for research that should highlight, in a more comprehensible manner, the impact on the survival of nutrition in some cancers. Although these anthropometric parameters already furnish prognostic discrimination and compare favorably in terms of reliability with DEXA-based assessment [23,45], they are quite imperfect. Thus, it is essential to develop and validate new techniques determining more precisely and in three-fold dimensions, the body reserves and their changes, with a special assessment of brown fat which negatively impacts survival [46]. In perspective, we have to move forward a new concept of functional body composition (FBC) to precise the specific phenotypes of patients with NSCLC which should be defined on physical performance tests, metabolism and inflammatory status, and evaluation of immune responsiveness.

## 4. Materials and Methods

This study is based on a retrospective analysis of a prospectively collected database. Demographic, morphomic, clinical and pathological data of 304 consecutive patients with NSCLC who underwent major resection (lobectomy or pneumonectomy) for NSCLC at Paris Center University Hospital in Paris, France, between 1 June 2001 and 31 December 2002 were analyzed. IRB approval was obtained (Comité de Protection des Personnes [CPP] Ile de France II, n° 2008-133 and 2012 06-12). A standard staging protocol was adopted. Patients surgically treated with sublobar resections (wedge or segmentectomies) were not included to avoid confounding factors. For stage IV disease (oligometastatic disease with ≤5 metastases) only lobectomy was proposed. Full nodal dissection was carried out in all cases. Adjuvant radiotherapy or chemotherapy was proposed on an individual basis following evidence-based discussions under the care of referring physicians. A centralized pathological review of the samples was performed. Tumor stages were reattributed in accordance to the 8th edition of the TNM classification [47].

### 4.1. Collected Data and Measurement of Low Muscle Mass

Patients’ characteristics, treatment procedures, and short-term outcomes were prospectively collected using a standardized case report form [48]. Long-term outcome was assessed by direct telephonic patient or family (in case of deceased patients) interviews. When no clinical follow-up was available, information on vital status was obtained through the patients’ municipality of birth.

Nutritional assessment was based on calculation of BMI and simple morphometry measurements. Current height (m) and weight (kg) were measured at surgical visit. Habitual (pre-disease) weight (defined as the higher patient’s self-measured weight in the last six months before diagnosis or onset of symptoms) was also recorded.

Weight variation was calculated and categorized as stable (0%), increasing (WI) (>1%), and loss (WL) (<1%). Three categories of WL were further determined: <5%, between 6% and 10%, and >10%.

Thus, pre-surgery and pre-disease BMI were derived according to the standard equation: BMI = weight (kg)/height (m^2^). Patients were categorized as follows: underweight (<18.5 kg/m^2^), normal weight (18.5 kg/m^2^ ≤ BMI < 25 kg/m^2^), overweight (25 kg/m^2^ ≤ BMI < 30 kg/m^2^) and obese (>30 kg/m^2^), according to the WHO definition [49].

Morphometric assessment of iTMM was based on measurements of mid-arm circumference (MAC) (in centimeters) and tricipital skin-fold thickness (TSF) (in millimeters) made at surgical visit. MAC was measured with a millimeter tape at the midpoint of the non-dominant arm, between the olecranon and the acromion. The fold-thickness measurements were made using a lipocalibrator (Holtain; Cambridge, UK). As many authors have done, low muscle mass was defined as a value below the 33rd percentile of iTMM of sex-specific populations [10,14,17,50].

To assess iTMM, the following indexes and values were calculated according to the equations [20]: mid-arm muscular perimeter (MAMP), mid-arm muscular area (MAMA), sex-corrected MAMA (scMAMA), total muscular mass (TMM), and iTMM: MAMP (cm) = MAC − [π × TSF]; $\mathrm{MAMA}\;\left({\mathrm{cm}}^{2}\right)\;=\;\mathrm{MAMP}2/4\pi$; scMAMA (cm^2^): Men = MAMA − 10, Women = MAMA − 6.5; TMM (kg) = height (cm) × [0.0264 + 0.0029 × scMAMA]; $\mathrm{iTMM}\;=\;\mathrm{TMM}/\mathrm{height}\;\left(\mathrm{kg}/m^{2}\right).$

Thus: (men)ITMM = 0.026 + 0.029 [{(MAC−[ π × TSF])²/4 π}−10] and (women)ITMM = 0.026 + 0.029 [{(MAC − [ π × TSF])²/4 π}−6.5]. iTMM was used for subsequent calculations. Low muscle mass was defined as iTMM less than the 33rd percentile of studied populations according to usual rules of the current literature and previous studies [10,14,16,17].

We applied the mid-arm measurement method because it is considered more accurate for iTMM calculation than that of calf circumference and better in obese patients that equations using height, weight, sex, age and ethnicity [20,24,51].

### 4.2. Data Analysis

Data processing and analysis were performed with the statistical Software SEM (SILEX Development, Mireffleurs, France). The results are expressed as the percentage, mean ±SD for normally distributed and median interquartile range for non-normally distributed quantitative variables.

Correlations were assessed by the Spearman rank test for continuous variables. Mann–Whitney and Kruskal–Wallis tests were used to perform group comparisons as appropriate. Percentage comparisons were performed by chi-square or Fisher exact test, as appropriate.

Survival analyses were performed by the Kaplan–Meier methods and curves were compared by the log-rank method. Factors associated with survival at univariable analysis (*p* < 0.05) were entered in step-wise multivariable models to assess their independent character. Because of strong interaction between parameters, we built three multivariable models, including, pre-disease BMI, pre-surgery BMI, and iTMM.

## 5. Conclusions

In conclusion, few morphomic parameters, easy to record clinically, strongly influence long-term survival of resectable NSCLC, independently of TNM. Thus, these markers should be integrated in the prognostic evaluation of patients undergoing surgery and in the follow-up. They may also serve as basis for assessment of body composition and changes after institution of nutritional supports. This new way of understanding cancer other than solely TNM staging, could lead to the development of new patient-directed strategies, including improvement of nutritional status and physical exercise, in the idea that restoring patient fitness, increasing weight and force, and improving immune function are essential to improve survival and reinforce the efficiency of curative treatments

## Figures and Tables

**Table 1:** Clinical, surgical and pathological characteristics related to morphometry in the cohort. SD: standard deviation. IQR: InterQuartile Range. COPD: Chronic Obstructive Pulmonary Disease. FEV1: Forced Expiratory Volume in 1 s. FVC: Forced Vital Capacity. ASA: American Society of Anesthesiologists classification of physical status.

Variable	*N* (%) or Mean ± SD or Median [IQR]
Men	254 (83.5%)
Age (range)	63 yrs (54–70 yrs)
Smoking	
Current and former smoking	286 (94.1%)
Smoking cessation for at least 2 months	177 (58.1%)
Cumulative smoking: Pack/Year index	40 (30–60)
Comorbid illnesses and Respiratory status	
Alcohol abuse	82 (26.3%)
Diabetes mellitus	41 (13.5%)
Ischaemic heart disease	43 (14.1%)
Stroke	16 (5.3%)
Lower limb atheroma	60 (18%)
Chronic bronchitis	198 (65.1%)
COPD	127 (41.8%)
FEV1 (% predicted)	80 (70–93.5)
FEV1/FVC (%)	72 (65–79)
ASA I–II/III/IV	6 (2%)/192 (63%)/103 (34%)/3 (1.0%)
Surgical procedures	
Lobectomy/bilobectomy	233 (76.6%)
Pneumonectomy	71 (23.4%)
Histological type	
Adenocarcinoma	131 (43%)
Non-adenocarcinoma	173 (57%)
Pathological stage and Tumor characteristics	
I	113 (37.2%)
II	87 (28.6%)
III	96 (31.6%)
IV	8 (2.7%)
Mean tumoral diameter	35 (25–50)
Vascular and lymphatic emboli	193 (63.5%)
Pleural invasion	160 (52.5%)
Morphomics and nutrional parameter	
Height	170 cm (165–175)
Pre-disease weight	72.5 Kg (63–82)
Pre-surgery weight	70 Kg (60.3–80)
% body weight variation	0% (0–6.75%)
stable body weight (*n* = 153)	0%
increase body weight (*n* = 16)	4% (2.25–8.75%)
decrease body weight (*n* = 135)	7% (4–12%)
Pre-disease body mass index (BMI)	24.9 Kg/m^2^ (22.5–27.3%)
Pre-surgery body mass index (BMI)	24.3 Kg/m^2^ (21.4–26.8%)
Mid-arm circumference (MAC)	26.9 cm +3.17
Tricipital skin-fold thickness (TSF)	11 mm (8–16)
Mid-arm muscular perimeter (MAMP)	23.1 cm + 3.2
Mid-arm muscular area (MAMA)	43.2 cm^2^ + 11.4
Sex-corrected MAMA (scMAMA)	33.75 cm^2^ + 10.8
Total muscular mass (TMM)	21.2 Kg ± 5.8
Indexed (on height) total muscular mass (iTMM)	7.3 Kg/m^2^ ± 1.7
(*n* = 147)	7.21 median (6–8.4); 6.49 (33rd percentile)

**Table 2:** Associations between clinical—pathological parameters and BMI or ITMM. The results are expressed in percentages, mean + SD or median [IQR], as appropriate. FEV1: forced expiratory volume in one second; ASA: American Society of Anesthesiologists classification of physical status. SD: standard deviation. IQR: InterQuartile Range. COPD: Chronic Obstructive Pulmonary Disease. FEV1: Forced Expiratory Volume in 1 s. ITMM: Indexed Total Muscular Mass. BMI: Body Mass Index.

Variable	Pre-Disease BMI ≤25 kg/m^2^	Pre-Disease BMI >25 kg/m^2^	*p*	Weight Increase	Stable Weight	Weight Decrease	*p*	Pre-Surgery BMI ≤25 kg/m^2^	Pre-Surgery BMI >25 kg/m^2^	*p*	ITTM ≤6.49 kg/m^2^	ITTM >6.49 kg/m^2^	*p*
Men	75.8	92.3	0.0001	75	88.8	78.5	0.038	77.7	22.5	0.00068	68	90.5	0.0008
Age (years)	59.8 ± 11.0	64.4 ± 9.5	0.0003	60.5 (50–66)	62 (53–69)	63 (55–71)	0.14	61.0 ± 11.0	63.5 ± 9.5	0.069	59.4 ± 10.8	63.1 ± 9.8	0.052
Smokers	91.9	96.5	0.09	100	95.4	91.8	0.5	92.9	95.8	0.30	93.6	94.7	0.91
Pack/year	40 (30–59)	40 (30–60)	0.75	50 (30–67)	40 (30–50)	40 (30–60)	0.6	40 (30–60)	40 (30–60)	0.82	40 (30–60)	43.5 (35–60)	0.55
Alcool abuse	30.4	23.1	0.15	37.5	24.8	28.1	0.63	28.3	25	0.53	29.8	22.1	0.32
Diabetes	11	16.3	0.19	18.7	11.9	14.7	0.96	14.3	12.4	0.65	8.9	18.1	0.16
Ischemic heart disease	10.1	18.4	0.09	13.3	18.5	94.7	1.0	19.0	20.9	0.72	8.7	12.9	0.47
Stroke	2.7	8.2	0.08	0	51.5	52.6	0.81	4.0	7.4	0.45	0	7.1	0.16
Lower limb atheroma	17.9	21.9	0.45	6.6	22.4	18.9	0.42	19.0	20.9	0.72	8.7	12.9	0.47
Chronic bronchitis	65.8	64.3	0.78	56.2	65.3	65.9	0.44	65.0	65.2	0.97	57.4	62.1	0.59
COPD	41.3	42.4	0.98	25	58.3	44.9	0.28	42.0	41.5	0.96	40	40.2	0.78
FEV1 (% predicted)	81.1 ± 18.1	88.7 ± 19.0	0.79	82.2 ± 23.2	80.7 ± 18.5	82.0 ± 18.3	0.97	81.2 ± 17.7	81.6 ± 20.0	0.83	78.2 ± 16.6	82.1 ± 21.7	0.25
ASA class I	2.5	1.4	0.7	0	2.6	1.5	0.68	2.2	1.7	0.8	0	3.2	0.18
ASA class II	64.6	60.8		75	63.6	60.4		63	62.5		76.6	60	
ASA class III	32.3	36.4		42.5	33.1	36.6		34.2	34.2		23.4	35.5	
ASA class IV	0.6	1.4		0	0.7	1.5		0.5	1.7		0	1	
Lobectomy and bilobectomy	79.4	73.4	0.22	87.5	81.6	69.6	0.23	75.4	78.3	0.56	76.1	74.8	0.86
Squamous Non-Squamous	58.8	52.5	0.045	46.6	55.4	46.4	0.38	56.9	53.4	0.018	56.8	54.3	0.3
Mean tumor diameter (mm)	40 (25–53)	35 (25–50)	0.82	37.0 ± 11.5	37.7 ± 18.1	44.1 ± 21.9	0.20	40 (26–55)	35 (25–50)	0.30	35 (25–51)	35 (25–50)	0.96
Pathologic stage I	31.4	43.7	0.063	37.5	44.4	29.1	0.07	46.2	31.2	0.039	30.4	38.9	0.058
Pathologic stage II	33.3	23.2		12.5	25.2	34.3		21	33.5		34.8	25.2	
Pathologic stage III	31.4	31.7		37.5	28.5	34.3		30.2	32.4		26.1	34.7	
Pathologic stage IV	3.8	1.4		12.5	2	2.2		2.5	2.7		8.7	1	
Emboli	66.9	59.8	0.22	73.3	63.6	62.3	0.4	65.1	61.3	0.52	63.4	64.4	0.91
Pleural invasion	59.3	45	0.014	40	48.9	58.0	0.18	60.1	41.0	0.0013	53.3	50	0.71

**Table 3:** Impact of clinical and pathological variables on survival. FEV1: forced expiratory volume in one second; ASA: American Society of Anesthesiologists classification of physical status. SD: standard deviation. IQR: Inter Quartile Range. COPD: Chronic Obstructive Pulmonary Disease. FEV1: Forced Expiratory Volume in 1 s. ITMM: Indexed Total Muscular Mass. BMI: Body Mass Index. 95% CI: 95%Confidence Interval.

Variable	5-Year Survival Rate (95% CI)	7-Year Survival Rate (95% CI)	*p* Value
Sex			
Men	45.4 (39.4–55.5)	37.7 (31.9–43.8)	
Women	51.9 (38.7–64.9)	42.3 (29.9–55.8)	0.056
Age			
≤65 years	52.2 (44.9–59.4)	42.5 (35.4–49.8)	
>65 years	38.7 (30.7–47.2)	32.9 (25.4–41.5)	0.017
Smoking			
Never or former smoker	49.4 (28.3–70.7)	49.4 (28.3–70.7)	
Current Smoker	46.7 (41.0–52.4)	38.1 (32.6–43.8)	0.57
Alcool			
Abuse	43.9 (33.7–54.7)	38.6 (28.7–49.5)	
No abuse	47.2 (40.8–53.7)	38.5 (42.3–45.0)	0.87
Diabetes mellitus			
Yes	41.7 (27.4–57.5)	38.9 (25.0–54.9)	
No	46.5 (40.4–52.7)	38.9 (33.0–45.2)	0.92
Angor			
Yes	44.8 (28.4–62.5)	41.4 (25.5–59.3)	
No	46.7 (39.6–54.0)	39.3 (32.5–46.6)	0.97
Stroke			
Yes	36.4 (15.2–64.6)	27.3 (9.7–56.6)	
No	47.0 (40.2–53.9)	40.3 (33.7–47.2)	0.17
Lower limb atheroma			
Yes	41.3 (28.3–55.7)	37.0 (24.5–51.4)	
No	48.3 (41.2–55.5)	39.3 (32.5–46.6)	0.47
Chronic bronchitis			
Yes	43 (36.0–50.3)	36.0 (29.3–43.3)	
No	52.2 (43.6–60.7)	42.4 (34.1–51.2)	0.15
COPD			
Yes	43.0 (34.6–51.8)	34.7 (26.8–43.4)	
No	49.3 (42.0–56.7)	40.9 (33.7–48.3)	0.33
FEV1 (% predicted)			
≥80	45.0 (37.4–52.9)	38.3 (31.0–46.2)	
<80	47.5 (39.5–55.6)	38.0 (30.4–46.2)	0.95
ASA score			
ASA I–II	51.2 (44.2–58.1)	42.2 (35.5–49.3)	
ASA III/IV	38.5 (29.8–48.1)	32.6 (24.3–42.0)	0.025
Resection type			
obectomy/bilobectomy	52.8 (46.4–59.1)	44.9 (38.6–51.3)	
Pneumonectomy	27.1 (18.3–38.2)	18.4 (11.1–28.9)	0.0001
Pathological type			
Adenocarcinoma	50.8 (42.8–58.6)	41.8 (34.1–49.8)	
Non -adenocarcinoma	44.1 (36.2–52.3)	37.5 (29.9–45.7)	0.17
Pathological stage			
I	68.4 (59.5–76.2)	55.6 (46.3–64.5)	
II	43.8 (33.9–54.3)	34.1 (24.9–44.7)	
III–IV	25.5 (18.2–34.6)	23.5 (16.4–32.4)	<0.0001
Mean tumoral diameter			
<3 cm	59.1 (49.0–68.6)	50.1 (40.1–60.2)	
≥3 cm	41.1 (34.8–47.8)	33.4 (27.4–40.0)	0.0011
Vascular/lymphatic emboli			
Yes	42 (35.0–49.4)	36.8 (30.1–44.2)	
No	57.1 (47.6–66.2)	43.4 (34.2–53.0)	0.06
Pleural invasion			
Yes	38.0 (30.8–45.7)	33.4 (26.5–41.1)	
No	57.0 (48.8–64.9)	44.6 (36.6–52.9)	0.0012

**Table 4:** Impact of morphomics on 5-year and 7-year survival rates in the whole cohort. BMI: Body Mass Index. 95% CI: 95%Confidence Interval.

Variable	5–Year Survival Rate (95% CI)	7–Year Survival Rate (95% CI)	*p*-Value
Pre-surgery BMI (kg/m^2^)			
<18.5	43.5 (25.6–63.2)	26.1 (12.5–46.5)	0.017
18.5–25	42.6 (35.2–50.4)	36.1 (29.0–43.9)	
25.01–30	45.8 (35.5–56.4)	38.5 (28.7–49.2)	
>30	70.3 (54.2–82.5)	61.2 (44.8–75.4)	
≤25	42.2 (35.3–49.4)	34.8 (28.3–42.0)	
>25	53.3 (44.4–62.0)	45.5 (36.8–54.5)	0.023
Pre-disease BMI (kg/m^2^)			
<18.5	41.7 (19.3–68.0)	16.7 (4.7–44.8)	0.036
18.5–25	42.0 (34.4–50.1)	34.9 (27.7–43.0)	
25.01–30	49.0 (39.6–58.5)	40.2 (38.3–49.9)	
>30	61.5 (45.9–75.1)	58.9 (43.3–72.8)	
≤25	42.0 (34.6–49.8)	33.5 (26.6–41.2)	
>25	52.4 (44.3–60.5)	45.2 (37.2–53.4)	0.021
Weight variation			
Stable	51.3 (43.5–59.0)	44.1 (36.5–51.9)	
Increase	68.8 (44.4–85.8)	55.6 (32.3–76.6)	
Decrease	39.0 (31.2–47.5)	31.1 (23.8–39.5)	0.0052
Pre–surgery height normalized total muscular mass (Kg/m^2)^			
(*n* = 147)	50.7 (39.3–62.0)	36.5 (26.3–48.2)	0.11
≤7.21 (median)	56.0 (44.4–67.0)	51.6 (40.1–62.9)	
>7.21			
≤6.49 (33rd percentile)	48.9 (35.3–62.8)	31.6 (20.1–46.0)	
>6.49	55.6 (45.5–65.2)	50.1 (40.2–60)	0.042

**Table 5:** Multivariate analysis of factors influencing survival (four different Cox models). n.s.: non-significant.

Variable	Relative Risk	95% CI	*p* Value
**Model 1**			
Age (years)			
≤65	1		
>65	1.45	(1.08–1.94)	0.0014
Resection type			
Lobectomy/bilobectomy	1		
Pneumonectomy	1.77	(1.27–2.47)	0.00083
ASA score			
I–II	1		
III/IV	1.56	(1.15–2.10)	0.0038
Stage			
I	1		
II	1.50	(1.25–1.81)	
III/IV	2.26	(1.56–3.28)	0.000018
Pre–disease BMI (Kg/m^2^)			
<25	1		
>25	0.66	(0.49–0.89)	0.006
Weight variation			
Increase	1		
Stable	1.36	(1.06–1.75)	
Decrease	1.86	(1.13–3.06)	0.014
**Model 2**			
Age (years)			
<65	1		
>65	1.42	(1.06–1.89)	0.019
Resection type			
Lobectomy/bilobectomy	1		
Pneumonectomy	1.75	(1.26–2.44)	0.00084
ASA			
I–II	1		
III/IV	1.54	(1.14–2.07)	0.0047
Stage			
I	1		
II	1.51	(1.25–1.81)	
III/IV	2.27	(21.57–3.29)	0.000012
Pre-surgery BMI (Kg/m^2^)			
<25	1		
>25	0.72	(0.54–0.98)	0.034
**Model 3**			
Age (years)			
<65			
>65			n.s.
Resection type			
Lobectomy/bilobectomy			
Pneumonectomy			n.s.
ASA			
I–II	1		
III/IV	1.72	(1.10–2.69)	0.018
Stage			
I	1		
II	1.57	(1.22–2.03)	
III/IV	2.47	(1.49–4.11)	0.00048
Pre–surgery height normalized total muscular mass (Kg/m^2^)			
≤6.49 (33rd percentile)	1		
>6.49	0.56	(0.37–0.87)	0.0091
